# Expression of Glycosaminoglycan Epitopes During Zebrafish Skeletogenesis

**DOI:** 10.1002/dvdy.23970

**Published:** 2013-04-05

**Authors:** Anthony J Hayes, Ruth E Mitchell, Andrew Bashford, Scott Reynolds, Bruce Caterson, Chrissy L Hammond

**Affiliations:** 1 Connective Tissue Biology Laboratory, Cardiff School of Biosciences and Cardiff Institute of Tissue Engineering and Repair, Cardiff UniversityCardiff, United Kingdom; 2 Departments of Biochemistry and Physiology and Pharmacology, University of BristolBristol, United Kingdom

**Keywords:** Zebrafish, craniofacial, development, extracellular matrix, proteoglycans, glycosaminoglycans, cartilage, bone

## Abstract

Background: The zebrafish is an important developmental model. Surprisingly, there are few studies that describe the glycosaminoglycan composition of its extracellular matrix during skeletogenesis. Glycosaminoglycans on proteoglycans contribute to the material properties of musculo skeletal connective tissues, and are important in regulating signalling events during morphogenesis. Sulfation motifs within the chain structure of glycosaminoglycans on cell-associated and extracellular matrix proteoglycans allow them to bind and regulate the sequestration/presentation of bioactive signalling molecules important in musculo-skeletal development. Results: We describe the spatio-temporal expression of different glycosaminoglycan moieties during zebrafish skeletogenesis with antibodies recognising (1) native sulfation motifs within chondroitin and keratan sulfate chains, and (2) enzyme-generated neoepitope sequences within the chain structure of chondroitin sulfate (i.e., 0-, 4-, and 6-sulfated isoforms) and heparan sulfate glycosaminoglycans. We show that all the glycosaminoglycan moieties investigated are expressed within the developing skeletal tissues of larval zebrafish. However, subtle changes in their patterns of spatio-temporal expression over the period examined suggest that their expression is tightly and dynamically controlled during development. Conclusions: The subtle differences observed in the domains of expression between different glycosaminoglycan moieties suggest differences in their functional roles during establishment of the primitive analogues of the skeleton. *Developmental Dynamics 242:778–789, 2013*. © 2013 Wiley Periodicals, Inc.

**Key Findings:**

- The developing zebrafish skeleton expresses many different glycosaminoglycan modifications.
- Multiple different glycosaminoglycan epitopes are dynamically expressed in the craniofacial skeleton.
- Expression of chondroitin sulfate moieties are dynamically expressed in the vertebral column and precede mineralisation.

## INTRODUCTION

Skeletogenesis is a dynamic and tightly coordinated process, both spatially and temporally, beginning as extracellular matrix (ECM) components are secreted and organised by cells and are later modified to suit their functional role in development, growth, and homeostasis (reviewed by Lefebvre and Bhattaram, 2010). In vertebrates, skeletogenesis occurs via two distinct mechanisms: (1) via intramembranous ossification, where bones develop directly, as occurs in the skull (reviewed by Franz-Odendaal, 2011); and (2) through endochondral ossification, where bone develops indirectly through a cartilage precursor (reviewed by Mackie et al., 2008, 2011). The majority of skeletal elements are formed via endochondral ossification: early cartilage templates are laid down during foetal development and are then progressively replaced with bone during post-natal growth. The cartilage provides the initial template for bone formation and then contributes to longitudinal growth of the bone via the epiphyseal growth plate (reviewed by Mackie et al., 2008, 2011). The cellular processes underlying these distinct mechanisms are orchestrated via a complex series of integrated, hierarchical signalling mechanisms involving hormones, morphogens, soluble growth factors, and cytokines, and must be tightly controlled at all levels to ensure coordinated development and growth of the skeleton as a whole (reviewed by de Crombrugghe et al., 2001; Chung et al., 2004; Adams et al., 2007; Mackie et al. 2008, 2011; Hojo et al. 2010).

Glycosaminoglycans (GAGs) are a major component of the developing skeleton and they play important roles in both its formation and function. GAGs are versatile and highly dynamic linear polysaccharides consisting of repeating disaccharide units that contain a hexosamine (i.e., glucosamine or galactosamine) and either a uronic acid (glucuronic acid or iduronic acid) or galactose. They include chondroitin sulfate (CS), dermatan sulfate (DS), keratan sulfate (KS), heparin/heparan sulfate (HS), and hyaluronan. With the exception of hyaluronan, all are covalently attached to a protein core via a tetrasaccharide linkage region to form proteoglycans (PGs) and all contain sulfates at various positions within their chains. The hexosamine, uronic acid, and galactose sugars in each disaccharide unit can be substituted with sulfate groups on the sugar hydroxyls in the 2, 4, and 6 positions through the activity of different sulfotransferase enzymes, thus generating considerable structural heterogeneity within individual GAG chains (Caterson, 2012). Sulfation confers a strong negative charge to the GAGs and allows them to bind water, conferring hydrodynamic strength to cartilaginous connective tissues. In addition, GAGs on PGs play important roles in regulating the development, growth, and homeostasis of skeletal tissues through their ability to interact with soluble, bioactive signalling molecules. Sulfation motif sequences within the chain structure of GAGs allow them to bind to diverse signalling molecules (e.g., growth factors, cytokines, chemokines, and morphogens) within the ECM and regulate their diffusion/sequestration/presentation in a highly specific manner. In this way, they can influence cellular behaviours such as proliferation, differentiation, and synthesis of ECM (Handel et al. 2005; Raman et al., 2005; Gandhi and Mancera, 2008; Caterson, 2012). Much is known of the functional role of HS on growth plate perlecan in this regard. The HS chains are involved in the binding and presentation of FGF2 to its cognate receptors thus regulating chondrocyte proliferation and affecting longitudinal bone growth (Govindraj et al., 2006). However, perlecan can only deliver FGF2 to its receptors after its CS chains have been removed, thus perlecan's CS chains allow it to function as a matrix reservoir for sequestration of FGF2 (Smith et al., 2007).

The importance of proper CS sulfation for normal skeletal development, morphology, and growth is slowly being recognised, and is underpinned by recent studies using chondrodysplastic animal models. In the brachymorphic (bm) mouse, for example, undersulfation of CS chains on growth plate PGs, caused by a mutation in the phosphoadenosine phosphosulfate synthetase (*Papss2*) gene, leads to altered Indian hedgehog signalling and changes in chondrocyte proliferation, manifesting as shortened limbs (Cortes et al. 2009). A similar correlation has been reported in diastrophic dysplasia (dtd) mice that are mutant for the sulfate transporter gene, *Slc26a2* (Gualeni et al. 2010). Chondroitin-4-sulfotransferase-1(C4S T-1)/ carbohydrate sulfotransferase 11 (CHST11), an enzyme involved in the 4-sulfation of chondroitin, has also recently been shown to modulate the activity of Wnt-3a, a soluble signalling molecule that plays key roles in skeletal development and morphogenesis (Klüppel et al., 2005; Klüppel, 2010; Liu et al., 2008; Nadanaka et al., 2011). The gene encoding this sulfotransferase enzyme (*C4ST-1/CHST11*) has also been identified as a major osteoarthritis susceptibility gene in a recent large genome-wide association study (arcOGEN Consortium and arcOGEN Collaborators, 2012). This combines with increasing evidence that osteoarthritis progression is likely underpinned by a reactivation of pathways that control the developmental process of endochondral ossification (reviewed by Loeser, 2010, Dreier R., 2010). Collectively, these observations highlight the growing importance of correct CS GAG sulfation in the development, growth, and homeostasis of skeletal tissues.

The zebrafish, *Danio rerio*, offers a number of advantages over the mouse for the study of vertebrate skeletogenesis (reviewed by Apschner et al., 2011; Hammond and Moro, 2012; Spoorendonk et al., 2010): it develops rapidly, can be maintained at low cost, and is easily amenable to genetic manipulation. Crucially, the developmental mechanisms underlying cartilage and bone growth in this organism also appear to be largely conserved with those of higher vertebrates (Yelick and Schilling, 2002; Knight and Schilling, 2006; Hammond and Schulte-Merker, 2009). An increasing number of zebrafish mutants defective in PG synthesis and/or GAG sulfation have now been shown to closely model human skeletal disease conditions (Wiweger et al., 2011). Zebrafish mutations in genes encoding enzymes essential for the synthesis of HS-PGs such as exostosin 2 (*ext2; dackel; dak*) and 3′-phosphoadenosine 5′-phosphosulfate transporter 1 (*Papst1; pinscher; pic*), model the human disease condition, Hereditary Multiple Exostoses (Clement et al., 2008, Wiweger et al. 2011). Mutations in other genes involved in PG synthesis and assembly, including glycosaminoglycan xylosylkinase (family with sequence similarity 20, member B; *fam20b*) and xylosyltransferase (*xylt1*), also result in profound downstream perturbations of chondrocyte maturation and endochondral ossification in zebrafish (Eames et al., 2011).Mutations in UDP-glucuronate decarboxylase 1 (*uxs1*), a gene instrumental in GAG attachment to core protein, meanwhile cause aberrant craniofacial morphogenesis in this animal model (Eames et al., 2010).

Whilst much is known of the molecular genetic mechanisms underlying zebrafish skeletogenesis, there are few studies that describe the GAG composition of its nascent ECM. The studies of Souza et al. (2007) and Zhang et al. (2009) have identified various classes of GAG from whole lysates of zebrafish using HPLC (high performance liquid chromatography) and mass spectroscopy. More recently Holmborn et al. (2012) demonstrated the presence of different sulfation motifs of CS and HS in zebrafish larvae 6 days post-fertilisation (dpf) by HPLC, and demonstrated that the sulfation levels vary in a variety of mutant lines. Nonetheless, large gaps remain in our understanding of the distinct GAG moieties that occur during the initial stages of skeletogenesis, when the primitive analogues of the skull and vertebral column are first established. This information would have considerable relevance to an improved understanding of the mechanisms underlying skeletogenesis. In this study, we describe the spatio-temporal expression patterns of major GAG moieties and CS sulfation isoforms in the craniofacial and axial skeletons of zebrafish larvae from 3 to 8 dpf.

## RESULTS AND DISCUSSION

### Skeletal Development of Larval Zebrafish (3–8 dpf)

Despite the growing importance of the zebrafish as a developmental model for vertebrate skeletogenesis, there are few studies describing the GAG composition of its skeletal elements during larval development (Kang et al., 2004; Eames et al., 2011; Holmborn et al., 2012). In this study, we have mapped the spatio-temporal distribution of a variety of GAG epitopes in the developing skeleton of larvae between 3 and 8 dpf, when the early cartilages of the skull and vertebral column are first established. The primarily cartilaginous tissues that we have examined have all been well studied (for example, Kimmel et al., 1995; Piotrowski et al., 1996; Schilling et al., 1996; Bird and Mabee, 2003; Haga et al., 2009) and show many parallels, from a developmental perspective, with those of evolutionarily more advanced species. We show that the GAG composition of the distinct skeletal tissues, and the subtly distinct expression patterns of CS sulfation that occur during zebrafish skeletogenesis, are of comparable complexity to those occurring in higher vertebrates.

**Fig. 1:**
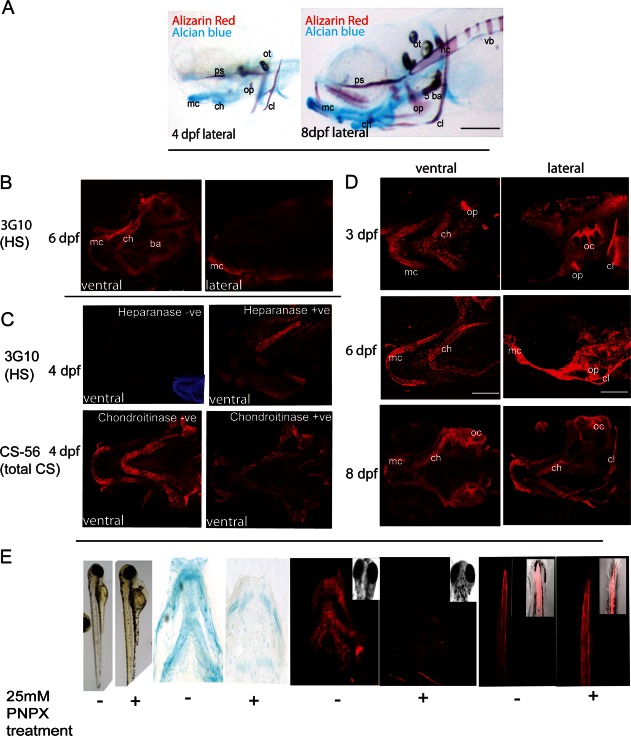
Chondroitin sulfate and Heparan sulfate are widely expressed in the developing zebrafish skeleton. **A:** Alcian blue- and alizarin red-stained skeletal preparations showing cartilage (blue) and mineralised tissue (red) at 4 and 8 dpf (lateral). **B:** Heparan sulfate labelling of the head with monoclonal antibody 3G10 at 6 dpf. **C:** Immunohistochemistry controls of 3G10 (labelling heparan sulphate) and CS56 (labelling native chondroitin sulphate), with and without chondroitinase ABC/heparitinase digestion as labelled at 4dpf. Heparitinase treatment is required to generate the epitope recognised by the 3G10 antibody, as such the heparitinase untreated fish show very limited immunoreactivity. CS56 antibody recognises a currently uncharacterised epitope present in native CS chains; treatment with chondroitinase ABC decreases immunoreactivity but doesn't completely prevent antibody binding. Ventral views with anterior to top. Inset in left-most panel with low levels/no labelling of antibodies show DAPI stained or brightfield images for orientation. **D:** Chondroitin sulfate labelling of the head from 3–8 dpf with monoclonal antibody CS-56. **E:** Treatment of larvae with the GAG chain inhibitor PNPX leads to decreased GAG synthesis and decreased labelling with CS-56 in newly synthesised cartilage elements, demonstrating that CS-56 specifically labels CS chains. Images are all at 4dpf after treatment with PNPX (controls with DMSO) from 50 hpf. Left panels: Brightfield views of whole larvae to show that while morphology is relatively normal heart oedema is present. Second pair of panels: Flat-mounted cartilages of the ventral jaw stained with Alcian blue to reveal GAG content. Treatment with PNPX leads to significant reduction in cartilage GAG levels. Third pair of panels: Confocal stacks of the central jaw of zebrafish labelled with CS-56 antibody at 4dpf, treatment with PNPX leads to a significant reduction in cartilage labelling of CS-56 such that levels are comparable with the reduction in GAG synthesis observed by Alcian blue labelling. Right pair of panels: Tail of the zebrafish labelled with CS-56, comparable labelling of the notochord is seen following treatment with PNPX, likely because notochord synthesis of GAGs occurs between 24 and 48hpf prior to the onset of treatment with PNPX. Insets in images with low levels/no labelling of antibodies show DAPI stained or brightfield images for orientation. mc, Meckel's cartilage; ch, ceratohyal; ba, branchial arches; op, operculum; ps, parasphenoid; oc, otic capsule; ot, otiliths; cl, cleithrum; 5ba, 5th branchial arch and teeth; nc, notochord; vb, developing vertebrae; ha, haemal arch; na, neural arch; sb, somite boundaries; +ve, positive; −ve, negative. Anterior is to left in all images. Scale bars = 100 μm in all panels.

The first skeletal elements to become visible during zebrafish development are the early cartilaginous elements of the skull and the most anterior aspect of the vertebral column. The zebrafish skull, like that of the human, is composed of cartilage and bone elements of both dermal and chondral origin and has been described in detail previously (Knight and Schilling., 2006). Histochemical staining of larvae with alcian blue (cartilage) and alizarin red (bone) showed that the cartilages of the lower jaw, the Meckel's cartilage, and the ceratohyal had all formed by 4 dpf, and a number of bone elements, all of dermal origin, were present, including the cleithrum, the operculum, and the mineralised otiliths (Fig. 1A). By 8 dpf, the head skeleton was well developed, containing multiple cartilage and bone elements including regions of mineralised tissue forming on a cartilage template, e.g., the ceratohyal and the 5th branchial arch (Fig. 1A). In contrast to the skull, the vertebral column becomes mineralised relatively late in development. Mineralised rings form directly around the notochord, first apparent at 7–8 dpf (Fig. 1A), and do not pass through a cartilage intermediate phase (Fleming et al. 2004). Correct patterning of these rings requires modulation of retinoic acid levels in the vertebral column (Spoorendonk et al., 2008). For clarity, the spatio-temporal expression patterns of the distinct GAG isoforms observed in the developing skull and vertebral column have been dealt with separately (below) and are summarised in Table 1.

**TABLE 1:** Summary of expression of GAG epitopes in developing skeletal elements^a^

Antibody name	Mc	Mx	Ch	Pq	Op	Cl	Nc	Ba	Hs	Oc	B r	Sb	Bn
(recognises)	(c)	(b)	(c)	(c)	(b)	(b)	(c)	(c)	(c)	(c)	(c)		
CS-56 (3dpf)	√	x	√	√	√	√	√	x	√	√	x	√	x
(total CS) (6dpf)	√	√	√	√	√	√	√	√	√	√	√	√	x
(8dpf)	√	√	√	√	√	√	√	√	√	√	√	√	x
3G10	√	x	√	√	x	x	x	√	√	√	x	x	x
(Heparan sulfate)													
1B5 (4dpf)	√	x	√	√	x	x	√	√	√	√	x	√	x
(C−0-S) (8dpf)	√	√	√	√	√	√	√	x	x	x	√	x	x
2B6 (4dpf)	√	x	√	√	x	x	x	√	√	x	x	√	x
(C-4-S/ DS) (8 dpf)		√	√		√	√	x	√	√	x	√	x	x
3B3+ (4dpf)	√	√	√	√	x	x	√	√	√	x	x	√	x
(C-6-S) (8dpf)	√	√	√	x	√	√	√	√	√	x	√	x	x
7D4 (4dpf)	√	x	√	√	x	x	x	√	√	√	x	x	x
(nativeCS/DS)8dpf)	√	x	√	√	x	x	√	√	√	√	x	x	x
5D4 (4dpf)	√	√	√	√	x	√	√	x	√	√	x	√	√
(KS) (8dpf)	√	√	√	√	√	√	√	√	√	x	√	√	√

a mc, Meckel's cartilage; pq, palatoquadrate; ch, ceratohyal; ba, branchial arches; op, operculum; ps, parasphenoid; oc, otic capsule; ot, otiliths; cl, cleithrum; 5ba, 5th branchial arch and teeth; nc, notochord; mx, maxilla; bn, brain; br, branchiosteal rays; hs, hyosymplectic; sb, somite boundary; bn, brain; (b), bone; (c), cartilage.

### Glycosaminoglycan Expression Patterns in the Developing Skull

#### Heparan sulfate

It is well known that HS chains on PGs play important roles in modulating signalling events during vertebrate skeletal development (Farach-Carson et al., 2005; Rodgers et al., 2008). We, therefore, studied the distribution of HS using a monoclonal antibody (3G10) that recognises a neoepitope occurring at the non-reducing terminus of all heparitinase-generated HS “stubs.” We observed HS to be broadly distributed throughout the developing skeleton at early developmental stages, with strong labelling occurring throughout the craniofacial cartilage elements (Fig. 1B). The presence of HS within these diverse connective tissues confirms that this GAG has involvement in zebrafish skeletogenesis, corroborated by loss of function studies in this organism (Wiweger et al., 2011). Loss of function of the various heparan sulfotransferases, for example, has been demonstrated to lead to abnormalities in diverse tissues and systems including muscle development (Bink et al., 2003), angiogenesis (reviewed by Stringer, 2006) and axon guidance (reviewed by Lee and Chien, 2004). Mutant zebrafish lines that have diminished HS levels, such as the exostosin 2 (*ext2; dackel; dak*) mutant, also show defects in chondrocyte stacking and aberrant craniofacial morphogenesis, underlining the key importance HS plays in skeletogenesis in this model (Clement et al., 2008; Wiweger et al., 2011). Indeed, the importance of HS, relative to other GAG moieties, in zebrafish skeletogenesis, has been highlighted in a recent analysis of the UDP-glucuronate decarboxylase 1 (*uxs1*) and beta-1,3-glucuronosyltransferase 3 (b 3gat3) mutant lines, where it has been shown that, in situations where the levels of PG linkage tetrasaccharides are reduced, HS biosynthesis is prioritised over CS biosynthesis (Holmborn et al., 2012).

#### Chondroitin sulfate

To establish the overall distribution of chondroitin sulfates between 3 and 8 dpf, we used a monoclonal antibody (mAb CS-56) that recognises an oligosaccharide sequence within generic native CS, but not DS chains (Ito et al., 2005) (Fig. 1C). CS immunolabelling was seen predominantly in ossifying cranial structures of both dermal and cartilaginous origin (Fig. 1A). At 2 dpf, weak staining was seen only in the cleithrum, a bone of dermal (or intramembranous) origin (data not shown). At 3 dpf, strong labelling was also present in the otic capsule, which forms the ear, and around chondrocytes forming the Meckel's cartilage and the ceratohyal. By 6 dpf, strong punctate CS immunolabelling was seen throughout the craniofacial cartilages. More uniform CS labelling was evident in bone elements such as the operculum, branchiosteal rays and cleithrum (Fig. 1C). At 8dpf, CS expression within the craniofacial skeleton was largely unchanged from that observed at 6dpf, with strong labelling occurring throughout all elements of the skull.

#### Chondroitin sulfation isoforms

**Fig. 2:**
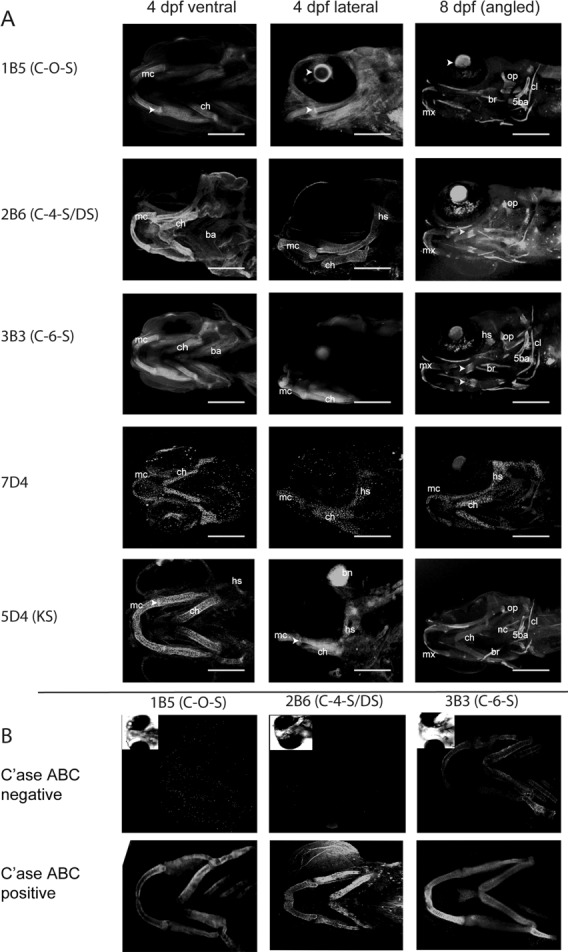
Various chondroitin sulfate moities are expressed in overlapping compartments during zebrafish skeletal development. **A:** Wholemount zebrafish heads labelled for various chondroitin and keratan sulfate epitopes with monoclonal antibodies 1B5, 2B6, 3B3, 7D4, and 5D4 (refer to Table 2, for antibody specificities). All images are confocal reconstructions presented with anterior facing to the left, and shown as ventral or lateral views. Scale bars = 100 μm in all panels. Triangular arrowheads in C-0-S and KS panels point to enrichment of GAG epitope in the jaw joint. Acute arrowheads in C-0-S panel point to expression in the lens. Acute arrowheads point to ossifying region of the ceratohyal. **B:** Chondroitinase ABC untreated controls show decreased/no immunoreactivity with the specific CS epitope antibodies. Treatment with chondroitinase ABC is required to generate the epitopes recognised by the 1B5 and 2B6 antibodies and, as such, chondroitinase untreated larvae show no immunoreactivity demonstrating that the antibodies are specific. The 3B3 antibody can also recognise an epitope present in native CS chains, so larvae untreated with chondroitinase ABC show changed rather than absent immunoreactivity with 3B3. mc, Meckel's cartilage; ch, ceratohyal; ba, branchial arches; op, operculum; cl, cleithrum; 5ba, 5th branchial arch and teeth; nc, notochord; mx, maxilla; br, branchiosteal rays; hs, hyosymplectic; C'ase, chondroitinase.

**Fig. 3:**
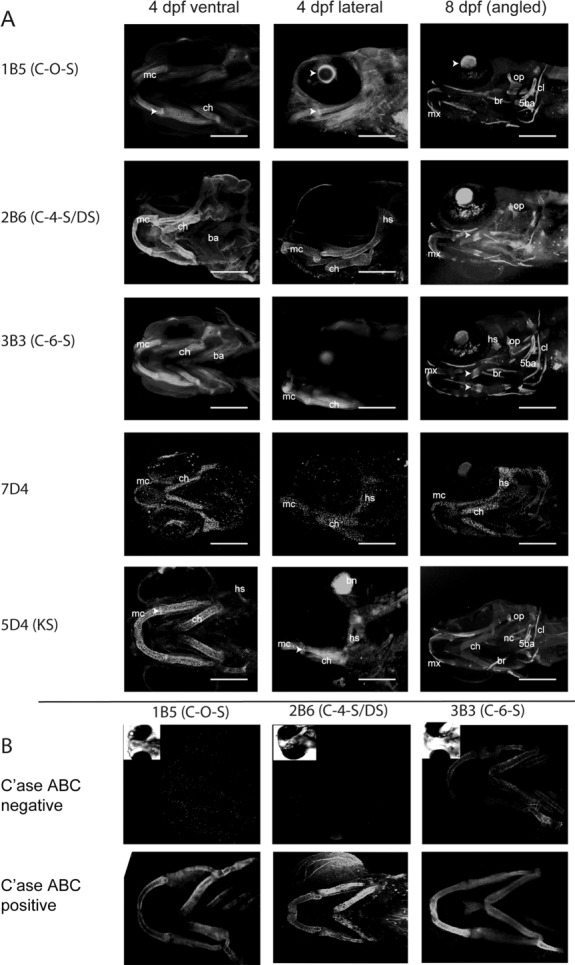
Chondroitin and keratan sulfate expression in the developing jaw. Confocal reconstructions showing comparative regions of the developing jaw of 8-day zebrafish larvae labelled with antibodies towards distinct chondroitin and keratan sulfate epitopes (refer to Table 2 for antibody specificities). In all images larvae are presented with anterior facing to the left. Arrowheads point to ossifying regions of the ceratohyal. ch, ceratohyal; mx, maxilla; mc, Meckel's cartilage; pq, palatoquadrate.

Using HPLC analysis, Holmborn and colleagues (2012) have recently shown that the PGs of 6-day-old zebrafish carry CS chains with a range of different sulfation patterns. It was estimated by the authors that approximately 34% of CS disaccharides are C-0-S, 28% C-4-S, and 36% C-6-S, with negligable amounts of C-2-S, C-6-S-4-S, C-4-S-2-S, and C-6-S-2-S (Holmborn et al., 2012). We have, therefore, utilised mAbs that specifically recognise 0-, 4-, and 6-sulfated isoforms of CS to elucidate their spatio-temporal expression patterns during early stages of skeletogenesis. In addition, we have investigated the presence of a unique, atypical, motif (with mAb 7D4), which occurs towards the linkage region of native CS/DS chains, and is expressed at sites of tissue differentiation and repair in many higher vertebrates (refer to Table 2 and Caterson, 2012). We found that all of the CS isoforms investigated were expressed over the developmental period (2–8dpf) in overlapping but subtly different domains of the developing craniofacial skeleton and surrounding connective tissues (Figs. 2 and 3). Significantly, the patterns of expression for each GAG moiety changed markedly between 4 and 8 dpf, suggesting that all are dynamically expressed during zebrafish skeletogenesis (see below).

**TABLE 2:** Antibodies Used in Immunohistochemistry^a^

Antibody (Dilution)	Monoclonal isotype	Pre-treatment	Specificity	Source/reference
7D4 (1:10)	IgM	K	Antibody recognizes a unique, as yet unidentified, native CS/DS sulfation motif epitope occurring towards the linkage region of GAG chain	Caterson (2012)
CS-56 (1:200)	IgM	K	Antibody reacts with an oligosaccharide sequence within generic CS, but not DS, chains.	Sigma Aldrich; Avnur and Geiger (1984); Ito et al. (2005)
1B5 (neat)	IgG1 kappa	ABC/K	Antibody recognizes a chondroitinase ABC -generated 0-sulfated CS ‘stub'.	Couchman et al. (1984)
				Caterson et al. (1985)
				Caterson (2012)
2B6 (neat)	IgG1 kappa	ABC/K	Antibody recognizes chondroitinase ABC-	Couchman et al. (1984)
			generated -4 sulfated CS/DS ‘stubs'.	Caterson et al. (1985)
				Caterson (2012)
3B3+ (1:10)	IgM,kappa	ABC/K	Antibody recognizes a chondroitinase ABC-generated -6 sulfated CS ‘stub'.	Caterson et al. (1985)
				Caterson (2012)
5D4 (1:20)	IgG1 kappa	ABC	Antibody recognizes a linear oversulfated oligosaccharide within native KS chains. Identifies both type I (corneal) and type II (skeletal) KS forms.	Caterson et al. (1983)
3G10 (1:100)	IgG2b	H/K	Antibody reacts with the unsaturated hexuronate (glucoronate) present at the non-reducing terminus of all heparitinase-generated HS ‘stubs'.	AMS biotechnology; David et al. (1992)

a ABC, chondroitinase ABC; CS, chondroitin sulfate; DS, dermatan sulfate; HS, heparan sulfate; K, proteinase K; KS, keratan sulfate; M, monoclonal; H, heparitinase.

#### Chondroitin-0-sulfate (C-0-S)

Immunofluorescent labelling with mAb 1B5 showed C-0-S to be expressed throughout the cartilaginous elements of the ventral jaw at 4 dpf. C-0-S was enriched in the region of the joint between the Meckel's cartilage and the palatoquadrate; arrowheads in Fig. 2) and was strongly expressed in the lens of the eye (acute arrowheads, Fig. 2). Weak expression was also seen in the brain. At 8 dpf, the expression of C-0-S in cartilaginous tissues became reduced (Figs. 2 and 3). However, strong expression was still detected in mineralising elements of the skull, including the operculum, cleithrum, and maxilla, in addition to continued expression within the lens (acute arrowheads in Fig. 2).

#### Chondrotin-4-sulfate/dermatan sulfate (C-4-S/DS)

After pre-treatment with chondroitinase ABC, mAb 2B6 detects both C-4-S, and its epimeric equivalent DS (refer to Table 2). C-4-S/DS was strongly expressed throughout the matrix of cartilaginous elements of the skull at 4 dpf, with no expression detected in non-cartilaginous tissues (Fig 2.). Unlike C-0-S, C-4-S/DS was not enriched in the jaw joint region. At 8 dpf, C-4-S/DS remained detectable throughout cartilaginous tissues, albeit at reduced levels; however, the strongest expression was seen in mineralising tissues, including dermal bone elements such as the operculum and the maxilla, but also in mineralising chondral elements, such as the ceratohyal (Figs. 2 and 3; arrowheads).

#### Chondroitin-6-sulfate (C-6-S)

Immunofluorescent labelling with mAb 3B3+ showed that C-6-S, like C-4-S/DS, was highly expressed throughout the matrix of cartilage elements at 4 dpf, although expression in the branchial arches was stronger for C-6-S than C-4-S/DS (Fig. 2). By 8dpf, C-6-S expression also became prominent mainly in mineralised elements of both dermal and chondral origin, thus having a similar expression pattern within the developing skull to that observed for C-4-S/DS (Figs. 2 and 3).

#### Atypical, native CS/DS chains

mAb 7D4 recognises a distinct, as yet unidentified, sulfation motif epitope within native CS/DS GAG chains that occurs towards the linkage region where the GAG is covalently attached to the PG core protein. This epitope is highly expressed across species in a range of connective tissues during their growth, differentiation, and attempted repair (Sorrell et al., 1990; Visco et al., 1993; Hayes et al., 2011; Caterson, 2012; Melrose et al., 2012). In contrast to the other CS epitopes, 7D4 was expressed exclusively within cartilaginous elements of the skull between 4 and 8dpf, and was not detected in bone or mineralising tissue (Figs. 2 and 3). Interestingly, the 7D4 expression pattern was similar to that produced by the total CS antibody (mAb CS-56), with punctate labelling occurring throughout the cartilage. The similarity in their labelling patterns may relate to an overlap in the epitopes detected by these antibodies within native CS chains (CS-56 does not recognise DS) that is lost following deglycosylation with chondroitinase ABC.

#### Keratan sulfate (KS)

At 4 dpf, KS was expressed in the cartilage elements derived from the first and second pharyngeal arches (the Meckel's cartilage and the ceratohyal). Like C-0-S, it was enriched around the jaw joint region (arrowheads in the two left panels for 5D4 of Fig. 2), but was only weakly detected in the branchial arches (Fig. 2). Strong expression was also seen in the brain when the fish were viewed laterally. At 8 dpf, KS remained detectable within the cartilage elements; however, like the various CS isoforms, its expression became strongest in mineralising tissues (Figs. 2 and 3; arrowheads).

### Chondroitin Sulfation Patterns in the Trunk and Developing Vertebral Column

**Fig. 4:**
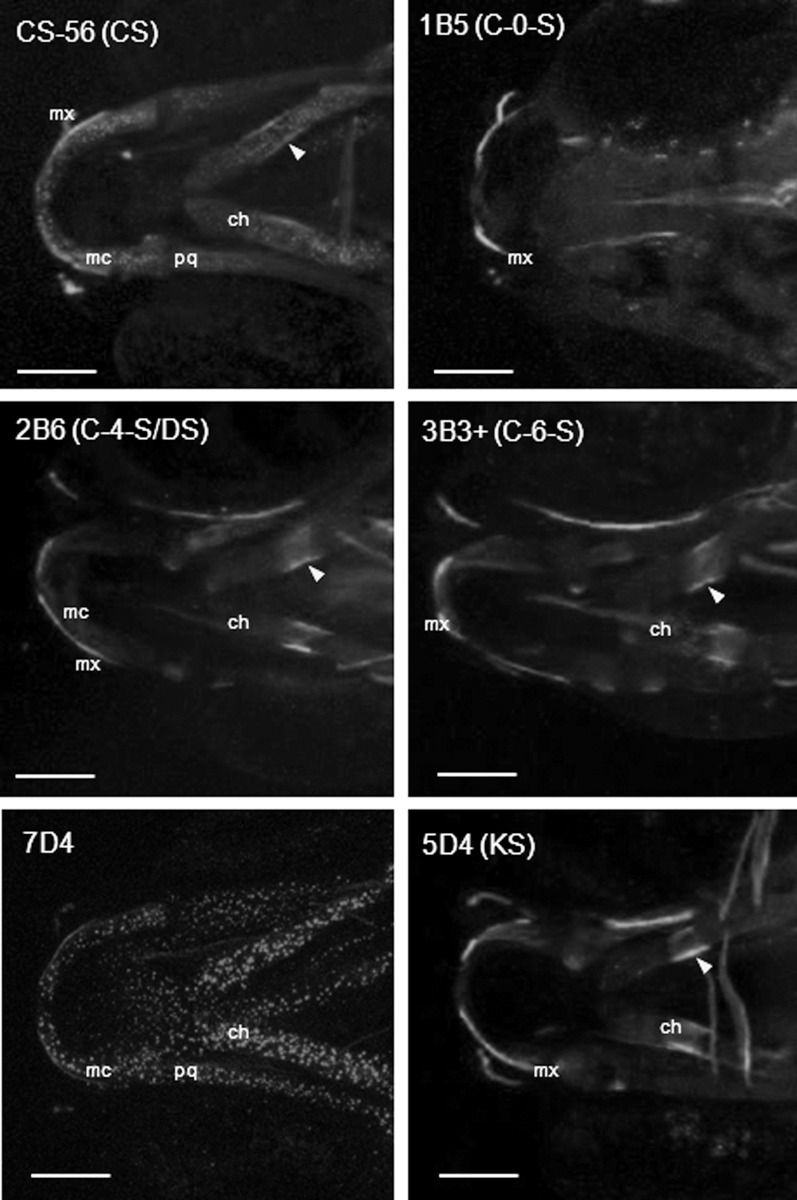
Chondroitin and keratan sulfate expression in the trunk and developing vertebral column. Immunohistochemistry of distinct chondroitin and keratan sulfate moities (as labelled) at 4 dpf and 8 dpf. All are lateral views with anterior to left. Trunk images at 4 dpf show a 4-somite span at the level of the cloaca. At 8 dpf, the images show an 8–9-somite span from the anterior end of the fish. Scale bars = 100 μm in all panels. hm, horizontal myoseptum; sb, somite boundary; sk, skin; nc, notochord; ha, haemal arch; na, neural arch; vb, vertebral body.

The spatio-temporal expression of generic CS, as identified by mAb CS-56, was somewhat more variable in the trunk and developing vertebral column than it was in the skull. CS was relatively widely expressed in the developing vertebral column, with the earliest expression seen surrounding the notochord at 3 dpf (data not shown). By 4 dpf, it was expressed more strongly around the notochord with fainter expression in the intersomite (Fig. 4). By 8 dpf, CS expression extended dorso-ventrally from the vertebral bodies to the sites of the future vertebral and haemal arches, which form a number of days later (Fig. 4, Spoorendonk et al. 2008). In summary, the expression of CS preceded mineralisation of cartilaginous elements throughout the axial skeleton.

The CS sulfation isoforms displayed striking differences in their patterns of expression within the vertebral column over the developmental period studied. C-0-S could not be detected in the notochord at 4 dpf, but expression was seen throughout the inter-somitic boundaries exhibiting a similar distribution to C-6-S (Fig. 4). In contrast, both C-4-S/DS chains and also atypical, native CS/DS (recognised by mAb 7D4) were absent from both these structures. KS has previously been shown to occur within the notochord of zebrafish larvae 24 hr post-fertilisation (Clement et al., 2008). In our study, KS expression was still evident within this structure at 4 dpf, as well as within the intersomitic boundaries (Fig. 4). By 8 dpf, C-0-S expression had diminished in the somites and was identifiable in a segmented pattern that corresponded to the mineralising rings surrounding the notochord (Figs. 1A, Fig 4). These ring structures were also identifiable at 8 dpf with mAbs 3B3+ and 7D4 indicating the presence of C-6-S and atypical, native CS/DS, respectively (Fig. 4). The extent to which the different CS/DS isoforms were expressed posteriorly along the vertebral column within these rings was variable. C-0-S was detectable within the first 8–12 rings; C-6-S, and atypical, native CS/DS chains (recognised by mAb 7D4), occurred only within the first 4–6 rings; whilst C-4-S/DS, was not detectable within the ring structures. Strong KS expression was also associated with the mineralising rings surrounding the notochord and also within the developing neural and hemal arches. KS was more widely distributed than the individual CS/DS sulfation isoforms and, in this regard, was more comparable with the total CS expression pattern.

The expression of different CS/DS and KS GAGs has been studied during the development of the vertebral column in a number of vertebrate species, including rat and human (Gotz et al., 1995; Hayes et al., 2011). In the rat, the different GAG isoforms display overlapping, but subtly-distinct, regionally-specific expression patterns within the constituent tissues of the spine, indicating that their synthesis is tightly and dynamically controlled to perform specific functional roles in development (Hayes et al., 2011). The results of the present study indicate that, with the exception of C-4-S/DS, a similar range of GAGs may perform similarly important developmental roles in the establishment of the axial skeleton of the zebrafish. The overlapping expression patterns of the different GAG moieties within the vertebral column, with C-0-S being most widely expressed, was particularly striking and appeared to correlate with the level of maturity of the cartilaginous vertebral elements, with increased levels of 6-sulfation in the more mature, anterior vertebral tissues. The specific role of differential expression of different GAG moieties during skeletal development is yet to be determined. Various factors, for example genetic drift and defence against pathogens, have been cited as potential reasons underlying the sheer diversity of GAG modification in vertebrates (Varki, 2011). Nonetheless, GAG sulfation remains a complex, energy-consuming process, and sulfation patterns within GAGs have been shown to encode molecular recognition and activity (Gama et al., 2006; Caterson, 2012). It is conceivable, therefore, that the different GAG isoforms may play subtly distinct functional roles in skeletogenesis.

GAGs occur in a wide range of musculoskeletal connective tissues including articular cartilage, bone, growth plate, muscle, and intervertebral disc, and their relative expression levels and distribution vary during development and maturation of skeletal tissues (Hardingham et al., 1994; Cheng et al., 1996; Bayliss et al. 1999; Hayes et al., 2001, 2011); following loading (Sauerland et al., 2003); and in disease (Hardingham, 1998), indicating the importance of maintaining the correct balance of these molecules, both spatially and temporally, throughout life. GAGs appear to influence the kinetics of collagen fibrillogenesis by modulating collagen fibril size and shape, and their aggregation into thicker bundles (Raspanti et al., 2008). GAGs also play important roles in the regulation of diffusion and presentation of growth factors, morphogens, and other soluble signalling molecules important in matrix growth and metabolism (Handel et al. 2005; Raman et al., 2005; Gama et al., 2006; Gandhi and Mancera, 2008; Caterson, 2012). The CS/DS chains on small leucine-rich PGs such as decorin and biglycan, for example, appear to play roles in both these respects (Hildebrand et al., 1994). Proteoglycans, together with integrins, are the main ECM adhesion receptors controlling growth factor signalling (Kim et al. 2011). Defective PG sulfation in the cartilage growth plate leads to defects in chondrocyte proliferation and maturation (Gualeni et al. 2010), while decreased CS sulfation leads to aberrant Indian Hedgehog signalling within the growth plate matrix (Cortes et al., 2009). There is increasing evidence that specific sulfation motif epitopes within the chain structure of GAGs can interact with growth factors, and different FGFs have been shown to have different sulfation-binding specificities (Gama et al., 2006; Shipp and Hseih-Wilson. 2007). Within the CS family, C-4-S levels control the diffusion of Wnt3a from producing cells to the target cells (Nadanaka et al. 2011) and regulate the balance between BMP and TGF-β signalling in developing growth plate (Klüppel et al., 2005; Klüppel, 2010). The dynamic expression patterns of different CS moieties that occur during zebrafish skeletogenesis may thus reflect the dynamic signalling events that are taking place during the establishment of the skeletal elements of the head and trunk.

The precise identity of the PG core protein to which each GAG moiety is affiliated remains unknown. CS is widely associated with a broad range of different PG species, whereas DS is found largely on versican, decorin, and biglycan, and HS on perlecan, the syndecans and glypicans (Kjellen and Lindahl, 1991). It is likely that the CS and KS GAGs, at least, may have a strong association with aggrecan, simply because this PG is typically the most abundant within cartilage and carries the greatest number of CS and KS chains (Watanabe et al., 1998). However, they may also occur on other species of PG in this organism. For instance, the zebrafish has been shown to contain other PGs including decorin (Zoeller et al., 2009), biglycan (Shintani et al., 2000), keratocan (Yeh et al., 2008), lumican (Yeh et al., 2010), and syndecans 2 and 4 (Chen et al., 2004; Whiteford et al., 2008) in addition to homologues of mammalian versican (dermacan; Kang et al., 2004). Furthermore, different species of PG may be substituted with different classes of GAG (Whiteford et al., 2008) thus imparting a further level of complexity.

In conclusion, the GAGs identified within the developing skeleton of the zebrafish show regionally specific, spatially restricted, and subtly different dynamic expression patterns. This indicates that in fish, as in higher vertebrates, GAG expression/sulfation is highly regulated during skeletogenesis to fulfill distinct functional roles.

## EXPERIMENTAL PROCEDURES

### Husbandry and Processing of Fish

Larval zebrafish, *Danio rerio*, raised as described by Westerfield (2000) were sampled at 3, 4, 6, and 8 dpf. A minimum of 30 fish were used for each developmental stage (n=3). Larvae were fixed in 4% PFA and stored in 100% MeOH at −20°C until required.

### Alcian Blue/Alizarin Red Staining of Wholemounts

Fixed larvae were rehydrated and then stained with alcian blue (cartilage) and alizarin red (bone) according to methods described by Walker and Kimmel (2007).

### Immunohistochemistry: Wholemounts

Stored larvae were rehydrated to PBS, washed in three changes of PBS containing 0.01% Tween 20 (PBST), permeabilised with proteinase K (10 μg/μl) in PBST for 25 min (3 dpf), 35 min (4 dpf), 50 min (6 dpf), or 80 min (8 dpf) at 37°C. To generate the 0-, 4-, and −6 sulfated antibody recognition sites for the CS neoepitope antibodies (i.e., mAbs 1B5, 2B6, and 3B3+), larvae were pre-digested with 0.5 U/ml chondroitinase ABC (Sigma-Aldrich, St. Louis, MO) in 100 mM Tris acetate buffer (pH 7.4) for 1 hr at 37°C. To generate the reactive HS neoepitope recognised by mAb 3G10, larvae were pre-digested with 5 mU/ml heparitinase (Amsbio, Lake Forest, CA) in 50 mM sodium acetate buffer (pH 7.0) containing 5 mM CaCl_2_ for 1 hr at 37°C, and re-fixed in 4% PFA. Larvae were blocked with 5% horse serum in PBST for 1 hr to prevent non-specific antibody binding and then incubated in primary antibody diluted in blocking serum overnight at 4°C. Larvae were labelled with monoclonal antibodies (mAbs; Table 2) recognising (1) native sulfation motif epitopes within chondroitin and keratan sulfate chains, and (2) enzyme-generated neoepitope sequences within CS (i.e., 0-, 4- and 6-sulfated isoforms) and HS GAG “stubs.” After the primary antibody labelling step, larvae were washed extensively in PBST, then incubated with a Dylight 550-conjugated goat anti-mouse secondary antibody for 2 hr at RT. The fluorescently-labelled larvae were washed and then imaged directly by confocal microscopy using a Leica SP5 confocal laser scanning microscope (Leica, Heidelberg, Germany).

### Antibody Labelling Controls

Antibody specificity controls consisted of: (1) replacing the primary antibody with buffer or naive immunoglobulin, (2) omitting the chondroitinase ABC enzyme digestion step for mAbs 1B5, 2B6, and 3B3+ and the heparitinase digestion step for mAb 3G10, and (3) pre-digesting larvae with chondroitinase ABC prior to labelling with mAb CS-56. Antibody controls showed (1) no non-specific labelling with either primary or secondary antibody; (2) loss of 1B5 and 2B6 (and altered 3B3; mAb 3B3 without chondroitinase, i.e., 3B3[-]), reacts with a terminal disaccharide present in native CS chains, i.e., a 6-sulfated galactosamine adjacent to a terminal glucuronate) (Hardingham, 1994) labelling following omission of the chondroitinase ABC pre-digestion step and loss of 3G10 labelling after omitting the heparitinase pre-digestion step, and (3) loss of CS-56 labelling following pre-digestion with chondroitinase ABC (refer to Figs. 1 and 2).

### Para-Nitrophenylxyloside Treatment

To further validate the GAG labelling patterns, GAG synthesis was perturbed by treating 50-hr zebrafish larvae with p-para-nitrophenyl-β-D xylopyranoside (PNPX), a competitive acceptor of CS/DS substitution on PGs. Treatment of larvae at earlier time-points resulted in lethality through cardiac oedema, presumably because CS/DS GAGs are critical for proper heart valve formation (Peal et al., 2009). Larvae were treated with PNPX (Sigma-Aldrich) twice daily at concentrations of 15, 20, and 25 mM, which was added to Danieau's water in which the larvae were grown. A minimum of 15 zebrafish larvae were used per dose sample. After 48 hr of treatment, the zebrafish larvae were fixed with 4% PFA, washed and stained with alcian blue and also immunolabelled with CS-56 to assess general GAG and CS distributions, respectively. Observation of larvae showed altered alcian blue staining and CS-56 labelling in a dose-dependent manner as anticipated, confirming the authenticity of the CS/DS labelling patterns (refer to Fig. 1).

## References

[b1] arcOGEN Consortium & arcOGEN Collaborators (2012). Identification of new susceptibility loci for osteoarthritis (arcOGEN): a genome-wide association study. Lancet.

[b2] Adams SL, Cohen AJ, Lassová L (2007). Integration of signaling pathways regulating chondrocyte differentiation during endochondral bone formation. J Cell Physiol.

[b3] Apschner A, Schulte-Merker S, Witten PE (2011). Not all bones are created equal: using zebrafish and other teleost species in osteogenesis research. Methods Cell Biol.

[b4] Avnur Z, Geiger B (1984). Immunocytochemical localization of native chondroitin-sulfate in tissues and cultured cells using specific monoclonal antibody. Cell.

[b5] Bayliss MT, Osborne D, Woodhouse S, Davidson C (1999). Sulfation of chondroitin sulfate in human articular cartilage. The effect of age, topographical position, and zone of cartilage on tissue composition. J Biol Chem.

[b6] Bink RJ, Habuchi H, Lele Z, Dolk E, Joore J, Rauch GJ, Geisler R, Wilson SW, den Hertog J, Kimata K, Zivkovic D (2003). Heparan sulfate 6-o-sulfotransferase is essential for muscle development in zebrafish. J Biol Chem.

[b7] Bird NC, Mabee PM (2003). Developmental morphology of the axial skeleton of the zebrafish, *Danio rerio* (Ostariophysi: Cyprinidae). Dev Dyn.

[b8] Caterson B (2012). Fell-Muir Lecture: Chondroitin sulphate glycosaminoglycans: fun for some and confusion for others. Int J Exp Path.

[b9] Caterson B, Christner JE, Baker JR (1983). Identification of a monoclonal antibody that specifically recognizes corneal and skeletal keratan sulfate. Monoclonal antibodies to cartilage proteoglycan. J Biol Chem.

[b10] Caterson B, Christner JE, Baker JR, Couchman JR (1985). Production and characterization of monoclonal antibodies directed against connective tissue proteoglycans. Fed Proc.

[b12] Chen E, Hermanson S, Ekker SC (2004). Syndecan-2 is essential for angiogenic sprouting during zebrafish development. Blood.

[b13] Cheng F, Heinegård D, Fransson L, Bayliss M, Bielicki J, Hopwood J, Yoshida K (1996). Variations in the chondroitin sulfate-protein linkage region of aggrecans from bovine nasal and human articular cartilages. 271.

[b14] Chung UI, Kawaguchi H, Takato T, Nakamura K (2004). Distinct osteogenic mechanisms of bones of distinct origins. J Orthop Sci.

[b15] Clement A, Wiweger M, von der Hardt S, Rusch MA, Selleck SB, Chien CB, Roehl HH (2008). Regulation of zebrafish skeletogenesis by ext2/dackel and papst1/pinscher. PLoS Genet.

[b16] Cortes M, Baria AT, Schwartz NB (2009). Sulfation of chondroitin sulfate proteoglycans is necessary for proper Indian hedgehog signaling in the developing growth plate. Development.

[b17] Couchman JR, Caterson B, Christner JE, Baker JR (1984). Mapping by monoclonal antibody detection of glycosaminoglycans in connective tissues. Nature.

[b18] David G, Bai XM, Van der Schueren B, Cassiman JJ, Van den Berge H (1992). Developmental changes in heparan sulfate expression: in situ detection with mAbs. J Cell Biol.

[b19] De Crombrugghe B, Lefebvre V, Nakashima K (2001). Regulatory mechanisms in the pathways of cartilage and bone formation. Curr Opin Cell Biol.

[b20] Dreier R (2010). Hypertrophic differentiation of chondrocytes in osteoarthritis the developmental aspect of degenerative joint disorders. Arthritis Res Ther.

[b21] Eames BF, Singer A, Smith GA, Wood ZA, Yan YL, He X, Polizzi SJ, Catchen JM, Rodriguez-Mari A, Linbo T, Raible DW, Postlethwait JH (2010). UDP xylose synthase 1 is required for morphogenesis and histogenesis of the craniofacial skeleton. Dev Biol.

[b22] Eames BF, Yan YL, Swartz ME, Levic DS, Knapik EW, Postlethwait JH, Kimmel CB (2011). Mutations in fam20b and xylt1 reveal that cartilage matrix controls timing of endochondral ossification by inhibiting chondrocyte maturation. PLoS Genet.

[b23] Fleming A, Keynes R, Tannahill D (2004). A central role for the notochord in vertebral patterning. Development.

[b24] Farach-Carson MC, Hecht JT, Carson DD (2005). Heparan sulfate proteoglycans: key players in cartilage biology. Crit Rev Eukaryot Gene Expr.

[b25] Franz-Odendaal TA (2011). Induction and patterning of intramembranous bone. Front Biosci.

[b26] Gama CI, Tully SE, Sotogaku N, Clark PM, Rawat M, Vaidehi N, Godard AA, Nishi A, Hsieh-Wilson LC (2006). Sulfation patterns of glycosaminoglycans encode molecular recognition and activity. Nature Chem Biol.

[b27] Gandhi NS, Mancerra RL (2008). The structure of glycosaminoglycans and their interactions with proteins. Chem Biol Drug Des.

[b28] Gotz W, Osmers R, Herken R (1995). Localisation of extracellular matrix components in the embryonic human notochord and axial mesenchyme. J Anat.

[b29] Govindraj P, West L, Smith S, Hassell JR (2006). Modulation of FGF-2 binding to chondrocytes from the developing growth plate by perlecan. Matrix Biol.

[b30] Gualeni B, Facchini M, De Leonardis F, Tenni R, Cetta G, Viola M, Passi A, Superti-Furga A, Forlino A, Rossi A (2010). Defective proteoglycan sulfation of the growth plate zones causes reduced chondrocyte proliferation via an altered Indian hedgehog signalling. Matrix Biol.

[b31] Haga Y, Dominique VJ, Du SJ (2009). Analyzing notochord segmentation and intervertebral disc formation using the twhh:gfp transgenic zebrafish model. Transgen Res.

[b32] Hammond CL, Moro E (2012). Using transgenic reporters to visualize bone and cartilage signalling during development in vivo. Frontiers Endocrinol.

[b33] Hammond CL, Schulte-Merker S (2009). Two populations of endochondral osteoblasts with differential sensitivity to Hedgehog signalling. Development.

[b34] Handel TM, Johnson Z, Crown SE, Lau EK, Proudfoot AE (2005). Regulation of protein function by glycosaminoglycans, as exemplified by chemokines. Annu Rev Biochem.

[b35] Hardingham T (1994). The sulphation pattern in chondroitin sulphate chains investigated by chondroitinase ABC and ACII digestion and reactivity with monoclonal antibodies. Carbohydrate Res.

[b36] Hardingham T (1998). Chondroitin sulfate and joint disease. Osteoarthritis Cartilage.

[b37] Hardingham TE, Fosang AJ, Dudhia J (1994). The structure, function and turnover of aggrecan, the large aggregating proteoglycan from cartilage. Eur J Chem Clin Biochem.

[b83] Hayes AJ, Benjamin M, Ralphs JR (2001). Extracellular matrix in development of the intervertebral disc. Matrix Biol.

[b38] Hayes AJ, Hughes CE, Ralphs JR, Caterson B (2011). Chondroitin sulphate sulphation motif expression in the ontogeny of the intervertebral disc. eCM.

[b39] Hildebrand A, Romarís M, Rasmussen LM, Heinegård D, Twardzik DR, Border WA, Ruoslahti E (1994). Interaction of the small interstitial proteoglycans biglycan, decorin and fibromodulin with transforming growth factor beta. Biochem J.

[b40] Holmborn K, Habicher J, Kasza Z, Eriksson AS, Filipek-Gorniok B, Gopal S, Couchman JR, Ahlberg PE, Wiweger M, Spillmann D, Kreuger J, Ledin J (2012). On the roles and regulation of chondroitin sulfate and heparan sulfate in zebrafish pharyngeal cartilage morphogenesis. J Biol Chem.

[b41] Hojo H, Ohba S, Yano F, Chung UI (2010). Coordination of chondrogenesis and osteogenesis by hypertrophic chondrocytes in endochondral bone development. J Bone Miner Metab.

[b42] Ito Y, Hikino M, Yajima Y, Mikami T, Sirko S, von Holst A, Faissner A, Fukui S, Sugahara K (2005). Structural characterization of the epitopes of the monoclonal antibodies 473HD, CS-56, and MO-225 specific for chondroitin sulfate D-type using the oligosaccharide library. Glycobiology.

[b43] Kang JS, Oohashi T, Kawakami Y, Bekku Y, Izpisua Belmonte JC, Ninomiya Y (2004). Characterization of dermacan, a novel zebrafish lectican gene, expressed in dermal bones. Mech Dev.

[b44] Kim SH, Turnbull J, Guimond S (2011). Extracellular matrix and cell signalling: the dynamic cooperation of integrin, proteoglycan and growth factor receptor. J Endocrinol.

[b45] Kimmel CB, Ballard WW, Kimmel SR, Ullmann B, Schilling TF (1995). Stages of embryonic development of the zebrafish. Dev Dyn.

[b46] Kjellen L, Lindahl L (1991). Proteoglycans: structures and interactions. Annu Rev Biochem.

[b47] Klüppel M (2010). The roles of chondroitin-4-sulfotransferase-1 in development and disease. Prog Mol Biol Transl Sci.

[b48] Klüppel M, Wight TN, Chan C, Hinek A, Wrana JL (2005). Maintenance of chondroitin sulfation balance by chondroitin-4-sulfotransferase 1 is required for chondrocyte development and growth factor signaling during cartilage morphogenesis. Development.

[b49] Knight R, Schilling T (2006). Cranial neural crest and development of the head skeleton. Adv Exp Med Biol.

[b84] Lee JS, Chien CB (2004). When sugars guide axons: insights from heparan sulphate proteoglycan mutants. Nat Rev Genet.

[b50] Lefebvre V, Bhattaram P (2010). Vertebrate skeletogenesis. Curr Op Dev Biol.

[b51] Liu F, Kohlmeier S, Wang C-Y (2008). Wnt signalling and skeletal development. Cell Signal.

[b52] Loeser RF (2010). Age-related changes in the musculoskeletal system and the development of osteoarthritis. Clin Geriatr Med.

[b53] Mackie EJ, Ahmed YA, Tatarczuch L, Chen K-S, Mirams M (2008). Endochondral ossification: How cartilage is converted into bone in the developing skeleton. Int J Biochem Cell Biol.

[b54] Mackie EJ, Tatarczuch L, Mirams M (2011). The skeleton: a multi-functional complex organ: the growth plate chondrocyte and endochondral ossification. J Endocrinol.

[b55] Melrose J, Isaacs MD, Smith SM, Hughes CE, Little CB, Caterson B, Hayes AJ (2012). Chondroitin sulphate and heparan sulphate sulphation motifs and their proteoglycans are involved in articular cartilage formation during human foetal knee joint development. Histochem Cell Biol.

[b56] Nadanaka S, Kinouchi H, Taniguchi-Morita K, Tamura J, Kitagawa H (2011). Down-regulation of chondroitin 4-O-sulfotransferase-1 by Wnt signaling triggers diffusion of Wnt-3a. J Biol Chem.

[b57] Peal DS, Burns CG, Macrae CA, Milan D (2009). Chondroitin sulphate expression is required for cardiac atrioventricular canal formation. Dev Dyn.

[b58] Piotrowski T, Schilling TF, Brand M, Jiang YJ, Heisenberg CP, Beuchle D, Grandel H, van Eeden FJ, Furutani-Seiki M, Granato M, Haffter P, Hammerschmidt M, Kane DA, Kelsh RN, Mullins MC, Odenthal J, Warga RM, NussleinVolhard C (1996). Jaw and branchial arch mutants in zebrafish II: anterior arches and cartilage differentiation. Development.

[b59] Raman R, Sasisekharan V, Sasisekharan R (2005). Structural insights into biological roles of protein-glycosaminoglycan interactions. Chem Biol.

[b60] Raspanti M, Viola M, Forlino A, Tenni R, Gruppi C, Tira ME (2008). Glycosaminoglycans show a specific periodic interaction with type I collagen fibrils. J Struct Biol.

[b61] Rodgers KD, San Antonio JD, Jacenko O (2008). Heparan sulfate proteoglycans: a GAGgle of skeletal-hematopoietic regulators. Dev Dyn.

[b62] Sauerland K, Plaas AH, Raiss RX, Steinmeyer J (2003). The sulfation pattern of chondroitin sulfate from articular cartilage explants in response to mechanical loading. Biochim Biophys Acta.

[b63] Schilling TF, Piotrowski T, Grandel H, Brand M, Heisenberg CP, Jiang YJ, Beuchle D, Hammerschmidt M, Kane DA, Mullins MC, van Eeden FJ, Kelsh RN, Furutani-Seiki M, Granato M, Haffter P, Odenthal J, Warga RM, Trowe T, Nüsslein-Volhard C (1996). Jaw and branchial arch mutants in zebrafish I: branchial arches. Development.

[b64] Shintani S, Sato A, Toyosawa S, O'hUigin C, Klein J (2000). Biglycan-like extracellular matrix genes of agnathans and teleosts. J Mol Evol.

[b65] Shipp EL, Hsieh-Wilson LC (2007). Profiling the sulfation specificities of glycosaminoglycan interactions with growth factors and chemotactic proteins using microarrays. Chem Biol.

[b66] Sorrell JM, Mahmoodian F, Schafer IA, Davis B, Caterson B (1990). Identification of monoclonal antibodies that recognize novel epitopes in native chondroitin sulfate glycosaminoglycan chains: their use in mapping functionally distinct domains of human skin. J Histochem Cytochem.

[b67] Smith SM, West LA, Govindraj P, Zhang X, Ornitz DM, Hassell JR (2007). Heparan and chondroitin sulfate on growth plate perlecan mediate binding and delivery of FGF-2 to FGF receptors. Matrix Biol.

[b68] Souza AR, Kozlowski EO, Cerqueira VR, Castelo-Branco MT, Costa ML, Pavão MS (2007). Chondroitin sulphate and keratan sulphate are the major glycosaminoglycans present in the adult zebrafish *Danio rerio* (Chordata-Cyprinidae). Glycoconj J.

[b69] Spoorendonk KM, Peterson-Maduro J, Renn J, Trowe T, Kranenbarg S, Winkler C, Schulte-Merker S (2008). Retinoic acid and Cyp26b1 are critical regulators of osteogenesis in the axial skeleton. Development.

[b70] Spoorendonk KM, Hammond CL, Huitema LFA, Vanoevelen J, Schulte-Merker S (2010). Zebrafish as a unique model system in bone research: the power of genetics and in vivo imaging. J Appl Ichthyol.

[b85] Stringer SE (2006). The role of heparan sulphate proteoglycans in angiogeneis. Biochem Soc Trans.

[b71] Varki A (2011). Evolutionary forces shaping the Golgi glycosylation machinery: why cell surface glycans are universal to living cells. Cold Spring Harb Perspect Biol 1.

[b72] Visco DM, Johnstone B, Hill MA, Jolly GA, Caterson B (1993). Immunohistochemical analysis of 3-B-3(-) and 7-D-4 epitope expression in canine osteoarthritis. Arthritis Rheum.

[b73] Walker MB, Kimmel CB (2007). A two-color acid-free cartilage and bone stain for zebrafish larvae. Biotech Histochem.

[b74] Watanabe H, Yamada Y, Kimata K (1998). Roles of aggrecan, a large chondroitin sulfate proteoglycan, in cartilage structure and function. J Biochem.

[b75] Westerfield M (2000). The zebrafish book. A guide for the laboratory use of zebrafish (*Danio rerio*.

[b76] Whiteford JR, Ko S, Lee W, Couchman JR (2008). Structural and cell adhesion properties of zebrafish syndecan-4 are shared with higher vertebrates. J Biol Chem.

[b77] Wiweger MI, Avramut CM, de Andrea CE, Prins FA, Koster AJ, Ravelli RB, Hogendoorn PC (2011). Cartilage ultrastructure in proteoglycan-deficient zebrafish mutants brings to light new candidate genes for human skeletal disorders. J Pathol.

[b78] Yeh LK, Liu CY, Chien CL, Converse RL, Kao WW, Chen MS, Hu FR, Hsieh FJ, Wang IJ (2008). Molecular analysis and characterization of zebrafish keratocan (zKera) gene. J Biol Chem.

[b79] Yeh LK, Liu CY, Kao WW, Huang CJ, Hu FR, Chien CL, Wang IJ (2010). Knockdown of zebrafish lumican gene (zlum) causes scleral thinning and increased size of scleral coats. J Biol Chem.

[b80] Yelick PC, Schilling TF (2002). Molecular dissection of craniofacial development using zebrafish. Crit Rev Oral Biol Med.

[b81] Zhang F, Zhang Z, Thistle R, McKeen L, Hosoyama S, Toida T, Linhardt RJ, Page-McCaw P (2009). Structural characterization of glycosaminoglycans from zebrafish in different ages. Glycoconj J.

[b82] Zoeller JJ, Pimtong W, Corby H, Goldoni S, Iozzo AE, Owens RT, Ho SY, Iozzo RV (2009). A central role for decorin during vertebrate convergent extension. J Biol Chem.

